# 肺癌伴胰腺转移42例临床分析

**DOI:** 10.3779/j.issn.1009-3419.2019.04.05

**Published:** 2019-04-20

**Authors:** 宇 张, 闽江 陈, 静 赵, 巍 钟, 燕 徐, 孟昭 王

**Affiliations:** 100730 北京，北京协和医学院，中国医学科学院，北京协和医院呼吸内科 Department of Respiratory Medicine, Peking Union Medical College Hospital, Chinese Academy of Medical Science and Peking Union Medical College, Beijing 100730, China

**Keywords:** 肺肿瘤, 胰腺转移, 急性胰腺炎, 梗阻性黄疸, 化疗治疗, 局部放疗, Lung neoplamsms, Pancreatic metastasis, Acute pancreatitis, Obstructive jaundice, Chemotherapy, Local radiotherapy

## Abstract

**背景与目的:**

多数肺癌患者在诊断时即出现远处转移，常见的转移部位包括肝脏、脑等，其中胰腺转移较为罕见。胰腺转移起病隐匿，预后普遍较差。目前医生对其的认识程度和重视程度不足。本研究旨在分析肺癌胰腺转移患者的病理特征、临床表现、治疗方案、预后以及相关因素对生存期的影响，探讨延长肺癌伴胰腺转移患者生存期及改善其生活质量的最佳治疗方案。

**方法:**

回顾性分析自1998年1月-2018年12月于北京协和医院就诊的42例信息资料完整的肺癌胰腺转移患者。

**结果:**

24例患者（57%）无特异性胰腺转移症状，18例患者（43%）出现胰腺转移相关症状，表现为腰背痛、急性胰腺炎、梗阻性黄疸。中位总生存期（overall survival, OS）为8.8个月。经多因素分析，存在急性胰腺炎等症状的患者预后偏差[危险比（hazard ratio, HR）=2.645，95%CI: 1.013-6.910，*P*=0.047]。胰腺转移后接受化疗的患者预后明显优于未接受化疗治疗的患者[HR=0.158, 95%CI: 0.049-0.512, *P*=0.002]。

**结论:**

肺癌胰腺转移罕见，预后差，存在胰腺相关症状者相对更差。接受化疗治疗能明显延长患者生存期。胰腺的局部放疗治疗可以缓解局部症状，利于患者进一步接受化疗治疗。有胰腺转移灶症状的患者可从化疗联合胰腺局部放疗的综合治疗中受益。

## Abstract (en)

**Background and objective:**

A number of patients with lung cancer have distant metastases at the time of diagnosis. The most common sites for metastases are liver, brain, *etc*. However pancreatic metastasis is relatively rare, with an insidious onset and poor prognosis. There are no sufficient recognition and attention of lung cancer with pancreatic metastasis. The aim of this study was to summarize the pathological characteristics, clinical manifestations, therapies and prognosis of pancreatic metastases of lung cancer, thus further exploring better managements for the best prolonged survival or quality of life.

**Methods:**

42 patients of lung carcinoma with confirmed pancreatic metastases hospitalized at the Peking Union Medical College Hospital from January 1998 to December 2018 were identified. We reviewed all medical documentations for complete information including diagnosis, treatment, prognosis features.

**Results:**

24 (57%) patients were asymptomatic or presented with non-specific symptoms. 18 (43%) patients had symptoms related to pancreatic metastases, such as acute pancreatitis, obstructive jaundice or pain of lumber back. The median overall survival (OS) was 8.8 months. Multivariate analysis suggested patients with symptoms had a poor prognosis compared with patients without pancreatic symptoms [(hazard ratio, HR)=2.645, 95%CI: 1.013-6.910, *P*=0.047]. Patients received chemotherapy had better prognosis versus those who did not [HR=0.158, 95%CI: 0.049-0.512, *P*=0.002].

**Conclusion:**

Pancreatic metastasis of lung cancer is rare and the prognosis is poor. Chemotherapy can prolong survival significantly. Local radiotherapy of the pancreas may alleviate local symptoms, improve quality of life, facilitate further systemic chemotherapy for patients to prolong survival. Patients with symptoms related to pancreatic metastases can benefit from the comprehensive treatment of chemotherapy combined with local pancreatic radiotherapy.

肺癌是全世界死亡率最高的癌症，多数患者确诊时已发生远处转移，如肺内、肝、骨、脑、肾上腺等，而胰腺是相对罕见的转移部位。肺癌患者出现胰腺转移比较隐匿，症状体征特殊，且预后普遍偏差。虽然已经有小细胞肺癌（small cell lung cancer, SCLC）或非小细胞肺癌（non-small cell lung cancer, NSCLC）转移至胰腺的个案报道和小样本的病例分析，但目前医生对其的认识程度和重视程度仍不足。当出现胰腺转移时提示患者已处于肺癌晚期，治疗方案以全身治疗为主，并辅助以胰腺的局部治疗，包括放疗等。本文回顾性分析了北京协和医院42例肺癌胰腺转移患者的临床表现、病理特征、胰腺转移后治疗方案及预后，探讨影响肺癌胰腺转移患者生存期的因素以及延长生存期，改善生活质量的治疗方案。

## 1 资料与方法

### 1.1 研究对象

检索1998年1月-2018年12月于北京协和医院诊治的肺部恶性肿瘤共17, 045例患者，以“胰腺转移癌”和“原发性支气管肺癌”为关键词，于我院病案室进行检索，共检索到62例患者，其中14例无法找到原始病例，1例病理证实为胰腺神经内分泌肿瘤，5例未获取原发肺癌病理，最终共入组肺癌伴胰腺转移患者42例进行分析。入组要求符合以下条件：①病理学证实的原发性肺癌，包括腺癌、鳞状细胞癌、腺鳞癌、小细胞肺癌、大细胞肺癌等；②疾病分期为Ⅳ期；③出现胰腺转移；④有详细的诊断和随访资料。因为回顾性病例研究，不涉及伦理和患者知情同意。

### 1.2 胰腺转移的诊断

胰腺转移具体诊断方法为：①首先影像学[胰腺增强计算机断层扫描（computed tomography, CT）、胰腺增强核磁或正电子发射计算机断层显像（positron emission tomography/CT, PET/CT）]提示胰腺占位，活检证实胰腺病灶病理为肺癌转移；②影像学（胰腺增强CT、胰腺增强核磁或PET/CT）提示胰腺占位，且在肺癌的诊治随访中胰腺病灶新发或增大；或经抗肿瘤治疗后胰腺病灶缩小；③肺癌诊断初期无胰腺转移证据，在肺癌诊治随访的过程中影像学（胰腺增强CT、胰腺增强核磁或PET/CT）提示胰腺占位；④影像学（胰腺增强CT、胰腺增强核磁或PET/CT）提示胰腺占位，且患者有胰腺占位的相关症状，包括急性胰腺炎、梗阻性黄疸或腰背痛。

### 1.3 患者临床资料收集

收集患者性别、年龄、临床症状（特别是胰腺占位的相关症状，包括急性胰腺炎、梗阻性黄疸或腰背痛）、病理类型、分期、胰腺检查的影像学资料、确诊胰腺转移的时间、疾病进展时间、全身和局部治疗情况。并通过病历系统和电话随访患者，随访终点为死亡。

### 1.4 统计学分析

采用SPSS 25.0和GraphPad Prism 7软件进行统计分析和作图。随访截止至2018年12月15日。总生存时间（overall survival, OS）定义为影像学或病理证实胰腺转移时开始至患者死亡。如果患者末次随访时仍存活，以影像学或病理证实胰腺转移时开始至末次随访时间记为删失值（censoring），如果患者末次随访时失访，以影像学或病理证实胰腺转移时开始至在医院记录的最后生存时间记为删失值。无进展生存期（progression-free survival, PFS）定义为患者出现胰腺转移时开始至有客观证据证实疾病进展（progressive disease, PD）、死亡或疾病尚未进展、失访的末次随访时间。采用*Kaplan-Meier*法计算中位生存时间并绘制生存曲线，并对患者性别、年龄、病理类型、胰腺转移与原发肿瘤是否同时诊断、胰腺转移灶症状、胰腺转移的位置、局部放疗、全身化疗对预后的影响分别进行单因素分析，统计学采用*Log-rank*检验。并将上述所有因素纳入*Cox*模型行多因素分析。*P* < 0.05为差异有统计学意义。

## 2 结果

### 2.1 患者一般情况

患者的一般情况见表 1。原发肺癌的病理类型均有组织病理或细胞学证实，其中SCLC患者18例（43%），NSCLC患者24例（57%），其中腺癌占20例，鳞癌占4例。胰腺转移的诊断中，符合①首先影像学提示胰腺占位，活检证实胰腺病灶病理为肺癌转移患者4例；②影像学提示胰腺占位，且在肺癌的诊治随访中胰腺病灶新发或增大或经抗肿瘤治疗后胰腺病灶缩小的患者13例；③肺癌诊断初期无胰腺转移证据，在肺癌诊治随访的过程中影像学（胰腺增强CT、胰腺增强核磁或PET/CT）提示胰腺占位14例；④影像学提示胰腺占位，且患者有胰腺占位的相关症状的患者18例（其中7例同时符合③④的标准）。

**1:** 肺癌胰腺转移患者42例的临床特点 Clinical characteristics of 42 cases

Clinical characteristics	*n*(%)
Gender	
Male	31 (73.8)
Female	11 (26.2)
Age (yr)	
≥65	12 (28.5)
< 65	30 (71.5)
Pathological type	
SCLC	18 (42.9)
NSCLC	24 (57.1)
Diagnosis time	
At initial	25 (59.5)
During the treatment	17 (40.5)
Site of pancreas	
Head	23 (54.8)
No head	19 (45.2)
Symptoms	
No	24 (57.1)
Yes	18 (42.9)
Local radiotherapy	
No	36 (85.7)
Yes	6 (14.3)
Chemotherapy	
No	9 (21.4)
Yes	33 (78.6)
SCLC: small cell lung cancer; NSCLC: non-small cell lung cancer.	

### 2.2 胰腺转移的相关情况

转移灶和原发灶同时诊断的患者25例；转移灶在抗肿瘤治疗后出现的患者17例，中位时间为11个月（范围5个月-60个月）。42例患者中，胰腺头部占位23例，体尾部占位21例，胰头及体尾部多发占位2例。18例患者存在胰腺转移灶相关症状，主要表现为急性胰腺炎10例，梗阻性黄疸6例，腰背痛6例，其中同时有急性胰腺炎和梗阻性黄疸症状4例。

### 2.3 胰腺转移后的治疗方案

#### 2.3.1 全身治疗

18例SCLC患者在发现胰腺转移后，17例接受了全身化疗，其中9例初诊即合并胰腺转移，接受一线化疗方案（依托泊苷联合铂类），8例诊治的过程中出现胰腺转移，接受二线化疗（拓扑替康或伊立替康）；1例因体能状态差，无法耐受化疗，仅接受支持治疗。

24例NSCLC患者中发现胰腺转移后，16例接受了全身化疗或化疗联合靶向药物治疗，其中12例为转移灶和原发灶同时诊断，化疗方案（即一线治疗方案）主要为紫杉醇/长春瑞滨/吉西他滨/多西他赛联合铂类（其中有6例患者联合靶向药物治疗，包括厄洛替尼、吉非替尼以及克唑替尼，3例患者存在基因突变），4例患者诊治的过程出现胰腺占位，胰腺转移后的主要治疗（即二线治疗方案）为长春瑞滨/吉西他滨联合铂类或培美曲塞单药治疗；1例患者仅接受艾维替尼靶向药物治疗，无表皮生长因子受体（epidermal growth factor receptor, *EGFR*）基因突变；7例患者未接受抗肿瘤治疗。

#### 2.3.2 局部治疗

18例SCLC患者中，4例患者接受胰腺局部放疗治疗，2例接受胆管支架置入术姑息性治疗。24例NSCLC患者中，2例患者接受局部放疗治疗，1例接受胆管支架置入术。

### 2.4 生存期

截止至2018年12月通过病历系统查阅及电话随诊，中位随访时间13.3个月，19例已死亡，9例存活，14例失访。中位PFS为4.2个月（95%CI: 1.634-6.766）。中位OS为8.8个月（95%CI: 3.986-13.614）（图 1）。

**1:**
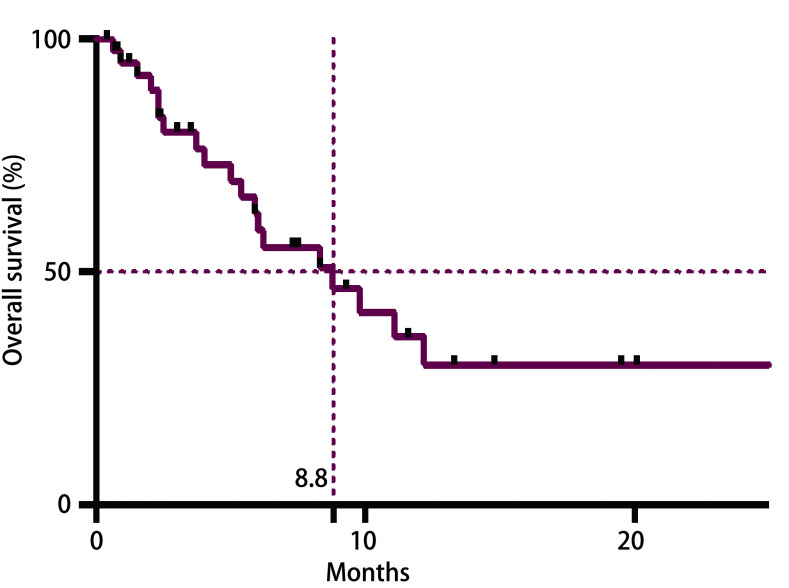
42例患者生存曲线 Survival curve of 42 lung cancer patients diagnosis with pancreatic metastases

### 2.5 患者生存的影响因素

#### 2.5.1 单因素分析

患者年龄、性别、病理类型、胰腺转移癌与原发肿瘤是否同时诊断（即是否初治）、胰腺转移部位、局部治疗对预后无明显影响（*P* > 0.05）。胰腺相关转移症状和全身化疗是影响患者预后的因素（*P* < 0.05）。

#### 2.5.2 多因素分析

将所有变量，即年龄、性别、病理类型、是否初治、胰腺转移部位、转移灶相关症状、化疗治疗、局部治疗引入Cox回归模型行多因素分析，发现全身化疗、胰腺占位的相关症状是影响总生存的独立因素。无胰腺转移相关症状的患者预后优于存在胰腺转移症状的患者（HR=2.645, 95%CI: 1.013-6.910, *P*=0.047）。胰腺转移后接受化疗的患者预后明显优于未接受化疗患者（HR=0.158, 95%CI: 0.049-0.512, *P*=0.002）（图 2）。

**2:**
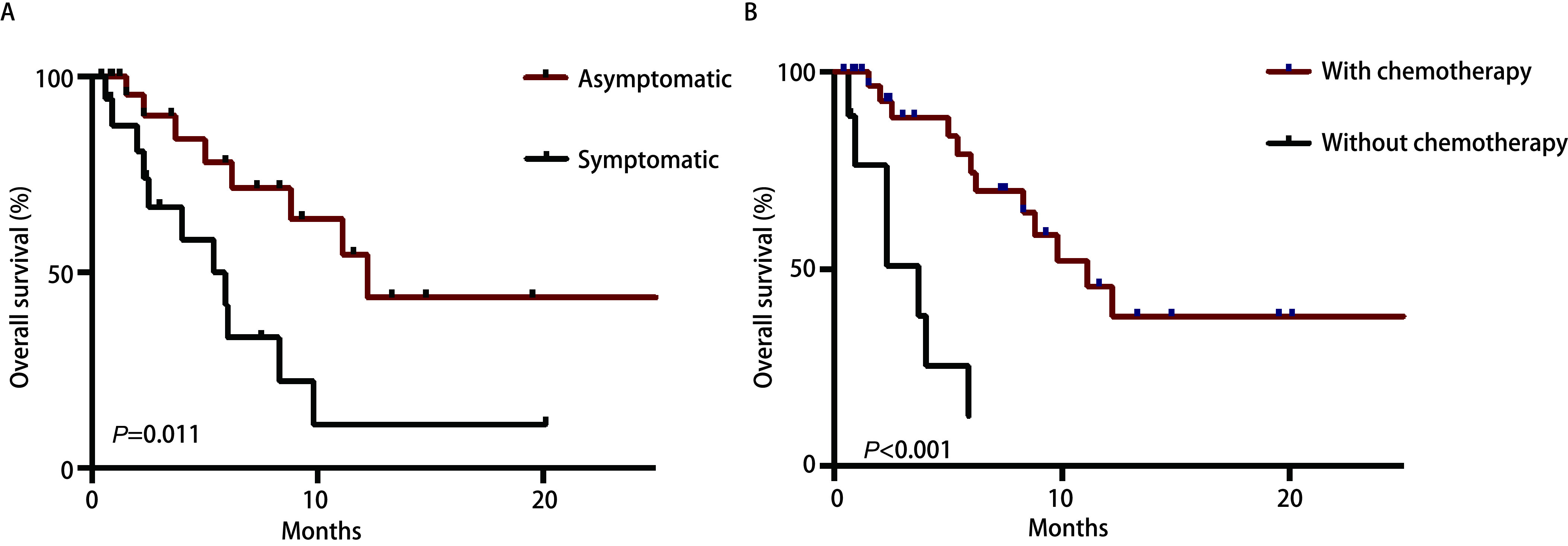
肺癌伴胰腺转移患者是否存在胰腺转移相关症状以及是否接受全身化疗治疗的生存比较。A：是否存在胰腺转移相关症状的生存比较；B：有无接受全身化疗的生存比较。 Survival curves between lung patients with pancreatic metastases but different symptoms and therapy. A: Survival curve showing the patients who presented with symptoms related to pancreatic metastases versus symptom-free; B: Curves showing the patients who underwent chemotherapy versus received no chemotherapy.

## 3 讨论

肺癌胰腺转移的发病率极低，在本组研究中仅占约0.25%（42/17, 045）。段建春等^[1]^曾对中国医学科学院肿瘤医院某科诊治的肺癌患者进行回顾性分析，胰腺转移的发病率约为0.69%（35/5, 016）。肺癌胰腺转移诊断的金标准是胰腺占位的病理组织或细胞学检查^[2, 3]^，但因该方法有创伤、风险较高，且部分患者因已处于疾病晚期，体能状态差，无法耐受胰腺病灶手术切除或经皮细针穿刺活检，或因无胰腺转移灶症状，活检意愿小，所以临床中仅有小部分肺癌胰腺转移通过胰腺肿物病理而确诊。大多数患者是以影像学检查为基础的临床诊断^[4]^。

大多数肺癌患者胰腺转移起病隐匿，少部分患者转移后出现腹痛、急性胰腺炎或梗阻性黄疸，极少数患者以急性胰腺炎和（或）黄疸为首发症状^[5-8]^。胰腺转移患者预后普遍较差，而目前医生对其的认识程度和重视程度不足，所以临床中对肺癌患者进行基线评估时或抗肿瘤过程出现上述症状时需高度警惕肿瘤侵犯胰腺的可能，避免漏诊。Adsay等^[9]^报道胰腺转移瘤中，最常见的原发肿瘤是肺癌，其次是胃肠道肿瘤和淋巴瘤。最常发生胰腺转移的肺癌病理类型是小细胞癌^[10, 11]^。本组42例肺癌胰腺转移患者中，SCLC占43%，比例较高；NSCLC占57%，其中腺癌为主，这可能与目前针对非鳞NSCLC的治疗手段进展迅速有关，在靶向治疗和免疫治疗等多种治疗方案下患者生存期较前明显延长，故在疾病逐渐进展过程中出现更多的远处脏器转移。Niu等^[12]^纳入2006年-2012年广东省肺癌研究所2, 872例NSCLC进行研究，193例出现罕见转移部位，如软组织、肾脏、胰腺等，与出现常见转移部位，如脑、肝脏等的患者进行对比，通过多因素分析确定罕见的转移部位为独立的不良预后因素。本组病例经多因素分析，认为患者存在胰腺转移灶相关症状、接受化疗治疗是独立预后因素。临床中选择改善生活质量、延长预后的治疗方案对患者很重要，尤其是当患者出现急性胰腺炎、梗阻性黄疸以及腰背痛等转移灶症状时，更应该个体化地选择最佳治疗方案。因SCLC对化疗高度敏感，对广泛期患者的治疗以全身性化疗为主，标准治疗方案为以铂类为基础的联合化疗，最常见的是铂类联合依托泊苷。晚期NSCLC癌患者的标准治疗亦是全身性治疗，根据肿瘤分子的特性，评估肿瘤组织有无体细胞驱动基因突变，包括*EGFR*基因突变、间变性淋巴瘤激酶（anaplastic lymphoma kinase, *ALK*）融合基因等，存在基因突变患者对相应靶向药物敏感，无驱动基因阳性（即*EGFR*基因突变以及*ALK*融合蛋白表达均阴性）患者主要应用免疫治疗和/或细胞毒化疗，具体取决于肿瘤程序性细胞死亡配体1（programmed death ligand 1, PD-L1）的表达和组织学^[13]^。本组24例NSCLC患者中，共16例患者接受化疗或化疗及靶向药物联合治疗，经多因素分析，接受化疗的患者生存期明显优于无化疗治疗患者，但在全身治疗同时需综合考虑患者年龄、体能状态、共存疾病等多因素进行个体化治疗，高龄患者和出现胰腺相关症状的患者体能状态较差，对化疗的耐受性差，可能无法从化疗中获益，所以给予局部放疗等姑息性治疗以改善患者症状，为患者继续化疗治疗提供机会，从而可以在一定程度上延长生存期^[10]^。本组18例SCLC患者中，接受胰腺局部放疗治疗4例，其中2例分别表现为腹痛、梗阻性黄疸，经过局部放疗后患者症状较前明显好转，并继续接受多程化疗治疗。接受胆管支架置入术后黄疸症状好转者2例。24例NSCLC患者中2例接受化疗联合胰腺放疗治疗。虽然本组研究中放疗治疗对患者预后无统计学意义，但因肺癌胰腺转移罕见，其中接受胰腺放疗治疗者更少，仍需进一步纳入患者进行分析研究。

综上所述，肺癌胰腺转移罕见，预后差，存在胰腺转移相关症状，如急性胰腺炎、梗阻性黄疸、腰背痛的患者预后更差。接受积极化疗治疗的患者生存期明显优于未接受化疗的患者。局部放疗治疗能缓解如急性胰腺炎等局部症状，改善患者体能状态，利于患者进一步接受化疗治疗，从而适当延长生存期。存在胰腺转移灶症状的患者可从胰腺局部放疗联合化疗的综合治疗中受益。在临床中，肺癌患者出现腹部症状时需格外关注有无胰腺转移，并及时调整最佳治疗方案以延长生存期。但目前对于无胰腺转移灶症状的患者在化疗基础上联合局部放疗是否可延长生存期仍有待进一步研究。
